# Acrylamide-based hydrogels with distinct osteogenic and chondrogenic differentiation potential

**DOI:** 10.1007/s40204-022-00196-5

**Published:** 2022-07-16

**Authors:** Z. M. Younus, P. Roach, N. R. Forsyth

**Affiliations:** 1 School of Pharmacy and Bioengineering, Keele University, Keele, UK; 2 Department of Chemistry, School of Science, Loughborough University, Leicestershire, UK; 3 Department of Clinical Laboratory Sciences, College of Pharmacy, University of Mosul, Mosul, Iraq

**Keywords:** Hydrogel, Osteochondral, Osteogenic, Chondrogenic, Mineralization

## Abstract

Regeneration solutions for the osteochondral interface depth are limited, where multi-material implants have the potential to delaminate affecting the regeneration process and impacting the final integrity of tissue interface. Here we explore regionally mixed hydrogel networks, presenting distinct chemical features to determine their compatibility in supporting osteogenic or chondrogenic cell behaviour and differentiation. Poly(*N*-isopropylacrylamide) (pNIPAM) and poly(*N*-*tert*-butylacrylamide) (pNTBAM) hydrogels were assessed in terms of their chemical differences, mechanical strength, internal architecture, porosity and capacity to support cell viability, migration, and differentiation. pNTBAM polymerized with a Young’s modulus of up to 371 ± 31 kPa compared to the more flexible pNIPAM, 16.5 ± 0.6 kPa. Viability testing revealed biocompatibility of both hydrogels with significantly increased cell numbers observed in pNTBAM (500 ± 95 viable cells/mm^2^) than in pNIPAM (60 ± 3 viable cells/mm^2^) (*P* ≤ 0.05). Mineralization determined through alkaline phosphatase (ALP) activity, calcium ion and annexin A2 markers of mineralization) and osteogenic behaviour (collagen I expression) were supported in both hydrogels, but to a greater extent in pNTBAM. pNTBAM supported significantly elevated levels of chondrogenic markers as evidenced by collagen II and glycosaminoglycan expression in comparison to little or no evidence in pNIPAM (*P* ≤ 0.05). In conclusion, structurally similar, chemically distinct, acrylamide hydrogels display variable capacities in supporting osteochondral cell behaviours. These systems demonstrate spatial control of cell interaction through simple changes in monomer chemistry. Fine control over chemical presentation during the fabrication of biomaterial implants could lead to greater efficacy and targeted regeneration of semi-complex tissues.

## Introduction

Advances in regenerative medicine and tissue engineering have resulted in substantial progress in identifying new approaches for the regeneration of osteochondral regions (Nukavarapu and Dorcemus 2013). Chemical and physical biomaterial characteristics define cellular responses where the physio-chemical properties and architectural construct specific to each material will determine the potential for cell attachment, proliferation, and differentiation (Wan et al. 2005; Murphy et al. 2010)^.^ Furthermore, material characteristics influence the cellular capacity to perform biological functions (Chen et al. 2018)^.^ As such, matching known parameters of the cell niche to those presented by biomaterials is essential in enabling some degree of control over cell behaviour.

A number of materials have had their capacity to support bone and cartilage tissue regeneration investigated including bio-ceramics, natural and synthetic polymers (Nukavarapu and Dorcemus 2013; Camarero-Espinosa and Cooper-White 2017). Of these, many are described as being supportive of osteogenic cell behaviour and mineralization e.g., hydroxyapatite (HA), polyglcolic acid (PGA), and polylactic acid (PLA) (Jones 2015; Bian et al. 2016). Promotion of chondrogenic behaviour is also reported for materials including hyaluronic acid and chitosan (Griffon et al. 2006; Malafaya and Reis 2009). It is further recognized that chondrogenesis may be enhanced further through surface modification with nano-topographical features or by manufacturing at nanoscale scaffold construct level (Izadifar et al. 2012). The presence of hydrophilic surface functional groups of the polymer may promote active osteoblast differentiation and activity (Chang and Wang 2011). Tailoring materials by alterations of the surface chemistry or incorporation of further cues can also impact on cell behaviour i.e., bio-active glass and carbon nano-tubes driving enhanced mineralization and osteogenic cell behaviour (Gajendiran et al. 2017).

The fabrication of materials for use in 3D culture systems enables a sophistication of architecture greater than that achievable with standard 2D culture alone. High water content of hydrogels and their tunable porous structures make them well-suited candidates for application across a number of tissues (Hoffman 2012). Acrylamide derivatives are often used as hydrogel materials due to their ease of fabrication ease of handling and tunable characteristics (Fang et al. 2019). Derivatives of these polymers, such as *N*-isopropylacrylamide (NIPAM) and *N-tert*-butylacrylamide (NTBAM) have been described as co-polymers with potential to stimulate tissue regeneration including that of bone and cartilage (Rzaev et al. 2007). This is due to their tuneable properties, achievable mainly through changing polymer concentration, solvent, or additives to control cross-linking, swelling and pore structure, with efforts made to present biomimetic features in cluding surface charge density, bio-active functional groups, and surface morphology (Lynch et al. 2005) NIPAM has also been widely used as a thermally responsive material, giving rise to changes in wettability, gel porosity and surface topography which can impact on cell adhesion porosity (Qavi et al. 2014). Surface micro-texture influences cell behaviour, including cell attachment and proliferation by providing features that mimic natural tissue micro-textures providing an appropriate environment for cell growth and development (Ermis et al. 2018; Flemming et al. 1999). Cell-surface guided differentiation has also been shown to be controlled through mechanical changes resulting from switch in material density (Helgeland et al. 2021)^.^

In the present work we make use of the small variation in molecular structures of NTBAM compared to NIPAM, with the former presenting an additional hydrophobic methyl group which has impacts on both the architecture of the polymer produced in solution-phase, as well as the chemical characteristics of the overall gel. This change has demonstrated controlled hindrance of cell attachment and protein adsorption when presented as a polymer film (Lynch et al. 2005). Here we demonstrate that pNIPAM and pNTBAM affect cell survival and behaviour differently, linking biological responses to 3D hydrogel structure and mechanical properties. Understanding how to utilize chemical changes in the fabrication of desirable materials for biological control will ultimately lead to advanced biomaterials. Making use of multiple materials within one hydrogel system provides a route for the development of semi-complex tissue constructs wherein multiple cell types are regionally held in proximity. Our example here using a partially mixed hydrogel system provides a demonstration of osteochondral tissue engineering.

## Materials and methods

All materials were used as received, with no additional purification or filtering. Chemicals were purchased from Sigma Aldrich unless otherwise stated.

### Preparation of hydrogels

Hydrogels were prepared by liquid phase atom transfer radical polymerization (Matyjaszewski 2012) using 8% w/v NIPAM monomer in RT dH_2_O. A cross linker *N,N′-*methylenebisacrylamide (MBA) was used to link the polymer network. Ammonium persulfate (APS) 10% w/v initiator was used alongside *N,N,N′,N′-*tetramethylethylenediamine (TMED) 2.6% v/v as an accelerator. NIPAM formed a transparent soft gelatinous structure requiring 2–3 min to form after rapid addition of the initiator. NTBAM was formed in a 1:1 combination of water and methanol heated to 37 °C. pNTBAM hydrogel formation required 10–15 min to completely gelate.

### SEM imaging

Hydrogels were observed using a bench-top Hitachi S4500 scanning electron microscope (SEM) at 5 kV. Hydrogels were frozen at – 20 °C, freeze dried, mounted onto carbon tape, and gold sputter coated. Pore size measurements were performed using Image J, with over 100 features measured per sample and 3 repeat samples measured.

### Water contact angle

Measurement of water contact angle was performed using a “Theta Lite Attension One Attension version 2.4” system. Samples were placed on a glass petri dish and compressed with a cover slip to acquire a flat surface while being dried at 70 °C in an oven. Water droplets, measured at 1 μL, were slowly placed onto test surfaces. Four measures were collected from each sample out of 4 samples.

### Mechanical compressive test

Compressive strengths were measured with the BOSE Electroforce system equipped with a 20 N loading cell and cross head speed of 0.05 mm/s. The samples were cylindrical in shape with dimensions of 4.5–5.0 mm height and 9.4–11.5 mm diameter. The load was applied until strain reached 90%. Compressive strength was determined from the maximum load of the applied stress–strain curve. Four samples of each hydrogel were tested, and an average obtained.

### Cell culture

A bone osteosarcoma cell line (MG63) and immortalized (human telomerase transcriptase (hTERT) transduced) chondrocytes (OK3H) (Dale et al. 2015) were both utilized to investigate the attachment and viability profile for the cell scaffold system. MG63 and OK3H were both cultured in Dulbecco’s Modified Eagle’s Medium (DMEM) containing 4.5 g/L glucose and sodium pyruvate and supplemented with 10% foetal bovine serum (FBS), and 2% *L*-glutamine. Culture environments were 37 °C in 5% CO_2_ culture incubators with media changed every 5 days. Primary human osteoblasts (hOB) and primary human chondrocytes (hCH) were obtained commercially from Promo Cell^®^ and cultured under the conditions described above. Routine trypsinization protocols were followed for cell passaging (Dale et al. 2015).

Differentiation was induced by addition of either osteogenic induction supplements (ascorbic acid 0.05 mM, *beta-*glycerophosphate 10 mM, dexamethasone 1 × 10^–5^ mM) or chondrogenic induction supplements (ascorbic acid 50 µM, dexamethasone 0.1 µM, *L-*proline 40 µg/mL, TGF-beta 3 and 10 ng/mL, and insulin-transferrin-selenium-ethanolamine 1% v/v).

### Viability and cell migration

Viability of cells was examined with the live/dead^®^ cytotoxicity/viability kit (Thermo Fisher Scientific L3224). The major components of the assay kit are calcein AM and ethidium homodimer-1 reagents. Calcein AM identifies the presence of live cells by detecting intracellular esterase activity and cell membrane integrity. Live/dead staining solution was prepared by mixing both reagents with PBS at the following rates; 1:200 of calcein AM and 1:50 of ethidium homodimer-1 in PBS. The cells viability profile was assessed after 21 days of cell culturing. The hydrogel samples were removed from media and washed 3 times with PBS. Next, these samples were incubated with 1 mL/sample of live/dead staining solution for 30 min at room temperature protected from light. Hydrogels were viewed under an Olympus U-TBI90 confocal microscope to observe the viable (green fluorescence) and the dead (red fluorescence) cells. The viability of cells upon hydrogel samples was identified by calculating the number of live and dead cells per specific regions of each sample. The number of live cells (green) and dead cells (red) were counted over a 1 mm^2^ area for a maximum of 5 regions of a captured × 4 microscopic images obtained for individual samples and the average was taken. The whole process was carried out using the cell counting tool of Image J software.

Cell migration was established after 21 days culture within the hydrogels. Hydrogel samples were removed from media, washed 3 times with PBS, and fixed in 10% paraformaldehyde for 30 min at room temperature. Then, each sample was incubated with 1 mL DAPI stain solution for 30 min at room temperature. Samples were then scanned across the z-axis using a 2 µm step size reaching a maximum of 150 slices of sample down from the top layer. The scanned distance for each sample was set to a maximum of 300 µm starting from the surface. Scanned files were processed via Image J software. The depth location was determined by nuclei localisation within the depth of the hydrogel.

### Immunostaining

Primary antibodies for collagens type I, II, and X (rabbit polyclonal antibodies, Abcam (cat. No. 34710, 34,712, and 58,632, respectively) confirmed differentiation. Visualization was enabled through appropriate secondary antibodies; Goat Anti-Rabbit IgG H&L, Abcam cat. No. 6718 and 6717, respectively). Hydrogel samples were fixed with 10% formaldehyde for 30 min at room temperature. Samples were blocked with 5% bovine serum albumin (BSA) in PBS for 2–3 h at 4 °C. This was followed by sample incubation with primary antibody solution overnight at 4 °C. The primary solution was prepared by mixing primary antibody (directed to type I, II, or X collagen) with 5% BSA in PBS at 1:200 ratio. The primary solution was then aspirated, and samples washed 4 times with 1% BSA in PBS solution. Samples were then incubated with secondary antibody (conjugated with either FITC or TRITC) in 5% BSA in PBS solution at 1:200 ratio at 4 °C for 4 h in the dark. Samples were then washed with 1% BSA in PBS followed by additional PBS washes. Nuclear staining was through DAPI stain for 30 min at room temperature followed by PBS washes. Hydrogels were imaged with an Olympus U-TBI90 laser fluorescent confocal microscope.

### Calcium mineralization and GAGs

Extraction of calcium minerals from hydrogel samples was performed using 0.5 M HCl solution. Hydrogels were first fixed with 10% paraformaldehyde in phosphate buffer saline (PBS) for 30 min at room temperature. Each hydrogel sample was incubated with 500 µL of HCl extraction solution at room temperature overnight on a rotary shaker. Calcium mineral was assessed using the calcium colorimetric assay kit (Sigma Aldrich MAK022) following manufacturer’s instructions with calcium ions present in samples (Morin 1974) detected and measured at 575 nm wavelength on a Synergy II BioTek plate reader.

GAGs matrix proteins were assessed by dimethylmethylene blue (DMMB) assay (Farndale et al. 1986). Samples GAGs content were obtained by incubating hydrogels with papain digestion buffer (prepared by dissolving 25 mg of papain from papaya latex in 50 mL PBS). Samples were freeze dried and then minced into small pieces with scalpels. Each sample was then digested with 500 µL papain digestion buffer, sealed with biofilm and incubated at 60 °C overnight. Sample lysates were then collected and mixed with DMMB working reagent and immediately examined for colour change at 525 nm by Synergy II BioTek plate reader.

### ELISA assessment of proteins

Matrix proteins indicatives for cell specific functions were assessed using sandwich ELISA assays (all from R&D systems). Hydrogels were digested in papain lysis buffer at 60 °C overnight. Cell lysates were then assessed for collagens I, II (utilizing Human Pro-Collagen I alpha 1 DuoSet and Human Pro-Collagen II DuoSet) and annexin A2 (utilizing Human Total Annexin A2 DuoSet) subsequent to the application of the assay protocol specific for each marker. Absorbance was measured with a Synergy II BioTek plate reader.

## Results

### pNIPAM and pNTBAM hydrogels have distinct architectural and mechanical characteristics

**Fig. 1:**
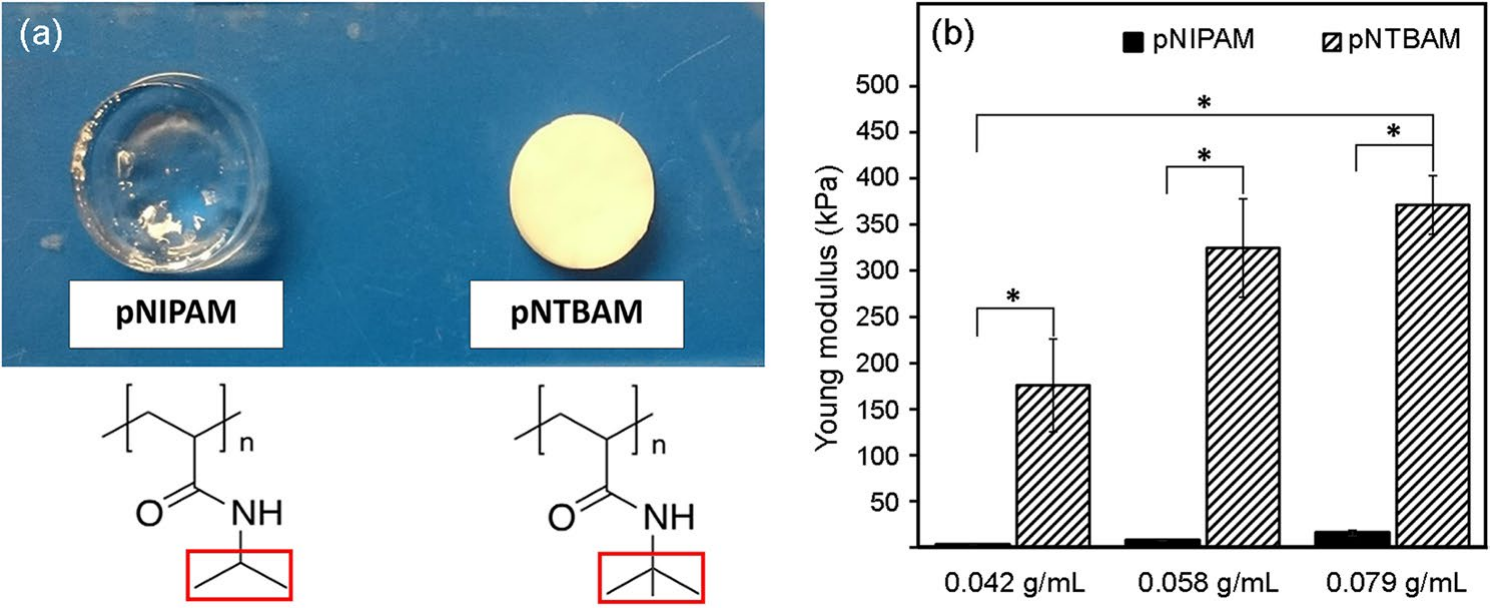
pNIPAM and pNTBAM general look and mechanical strength. **A** Physical appearance of hydrogels following gelation. Chemical structure is indicated and distinct chemical functional groups are indicated. **B** Young’s modulus as a measure of hydrogel stiffness. Data presented as kPa for each hydrogel and monomeric subgroups. Asterisk indicates *P* ≤ 0.05. Data presented as mean ± SD, *n* = 4

Polymerization resulted in pNIPAM and pNTBAM hydrogels that were immediately distinguishable through differing colour and texture (Fig. 1A). pNIPAM formed a translucent flexible material while pNTBAM formed a white, rigid, and easy to handle mass. pNIPAM flexibility and elasticity was immediately evident following on from stress-induced deformation with a return to normal shape after releasing compression. pNTBAM on the other hand retained a deformed profile after removal of compression. Stiffness profiling indicated significantly higher stiffness for pNTBAM compared with pNIPAM (*P* ≤ 0.05) (Fig. 1B). pNTBAM resisted a force ≥ 25 N whereas a maximum of 12 N was observed for pNIPAM at the same strain level (90%).

**Fig. 2:**
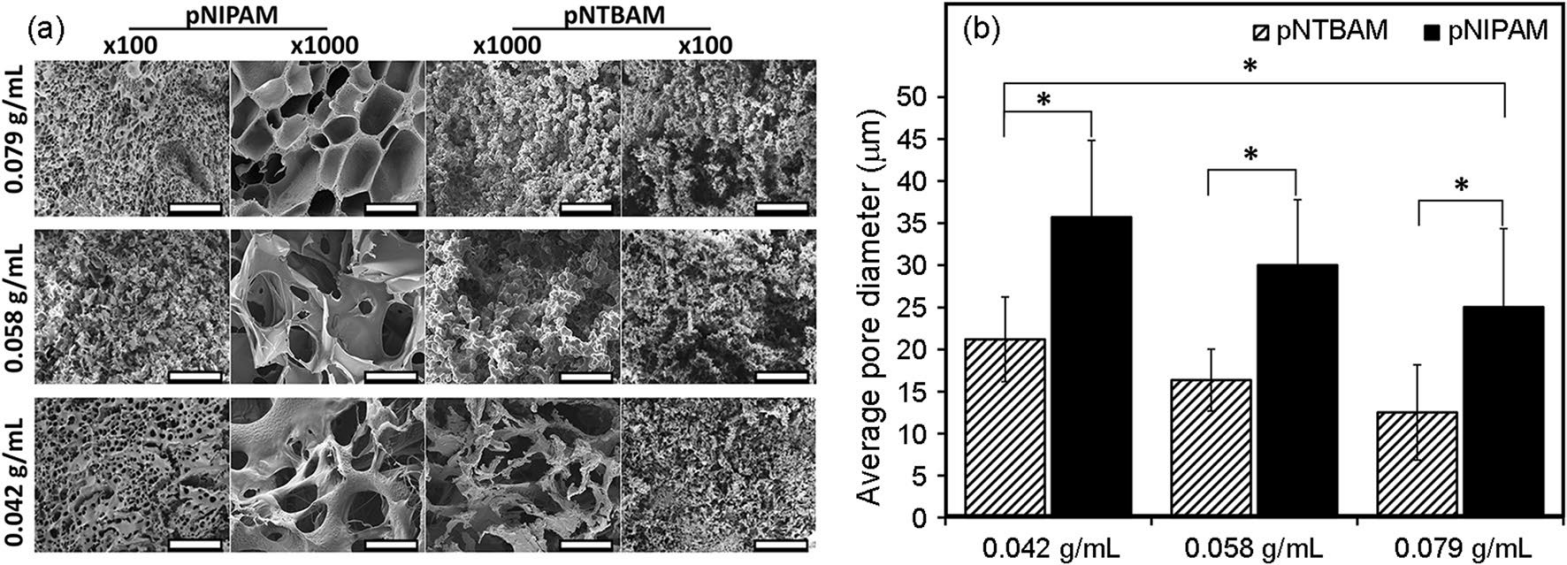
Hydrogels’ interior design and pore characteristics. **A** SEM imaging of pNIPAM and pNTBAM determining internal architectural of monomeric subgroups. Image capture at 100× and 1000× magnification for each monomeric concentration. Scale bar 300 µm for × 100 and 30 µm for the × 1000 images. **B** Pore size comparison between pNIPAM and pNTBAM hydrogels’ subgroups. Pore diameter indicated in µm following analysis in ImageJ software.Asterisk indicates *P* ≤ 0.05. Data presented as mean ± SD, *n* = 4

Consistent with their distinct physical properties SEM revealed internal hydrogel architectural differences with both hydrogels having porous matrices that differed in both pore size and shape (Fig. 2A). Pore diameter measurements revealed significantly larger mean pore size for 0.079 mg/mL hydrogel concentration subgroups of pNIPAM, 25 ± 9 µm versus pNTBAM, 12.5 ± 5 µm (*P* ≤ 0.05). Pore size increased with lowering hydrogel monomeric concentrations for both hydrogels (*p* ≤ 0.05). For pNIPAM, 0.058 g/mL and 0.04 g/mL concentration resulted in mean pore diameters of 29.9 ± 7.7 µm and 35.7 ± 9 µm, respectively. Similarly, with pNTBAM, 0.058 g/mL and 0.04 g/mL concentrations resulted in mean pore diameters of 16.3 ± 3.6 µmand 21.2 ± 5 µm, respectively (Fig. 2B).

**Fig. 3:**
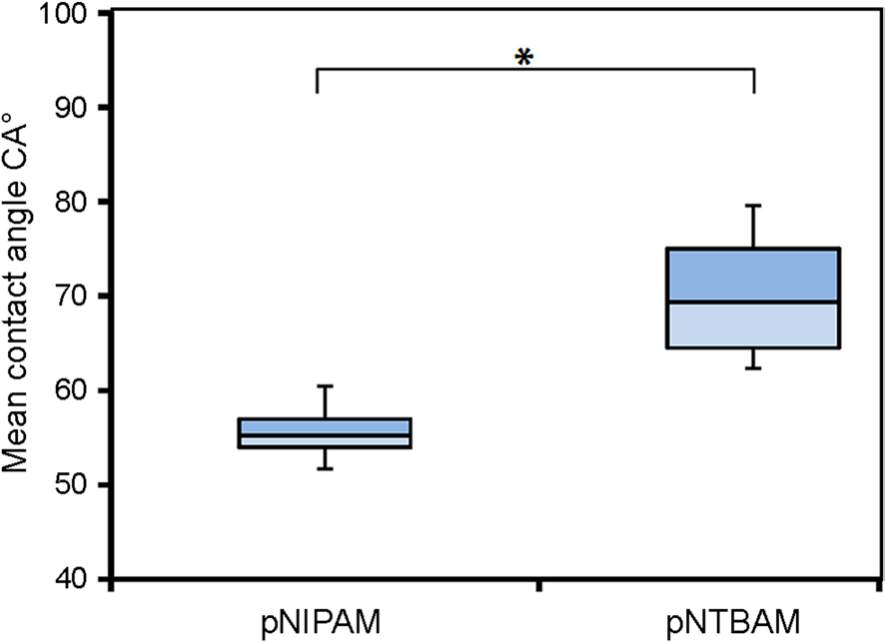
Wettability of pNIPAM and pNTBAM. The water contact angle records for pNIPAM and pNTBAM polymers showing significant difference between polymers. *P* ≤ 0.05 results correspond to mean ± SD, *n* = 4

Wettability of the gels by water contact angle measurements of dried materials demonstrated clearly the change in hydrophobicity of the pNTBAM gel compared to pNIPAM (Fig. 3).

### pNIPAM and pNTBAM promote distinct cell behaviour

**Fig. 4:**
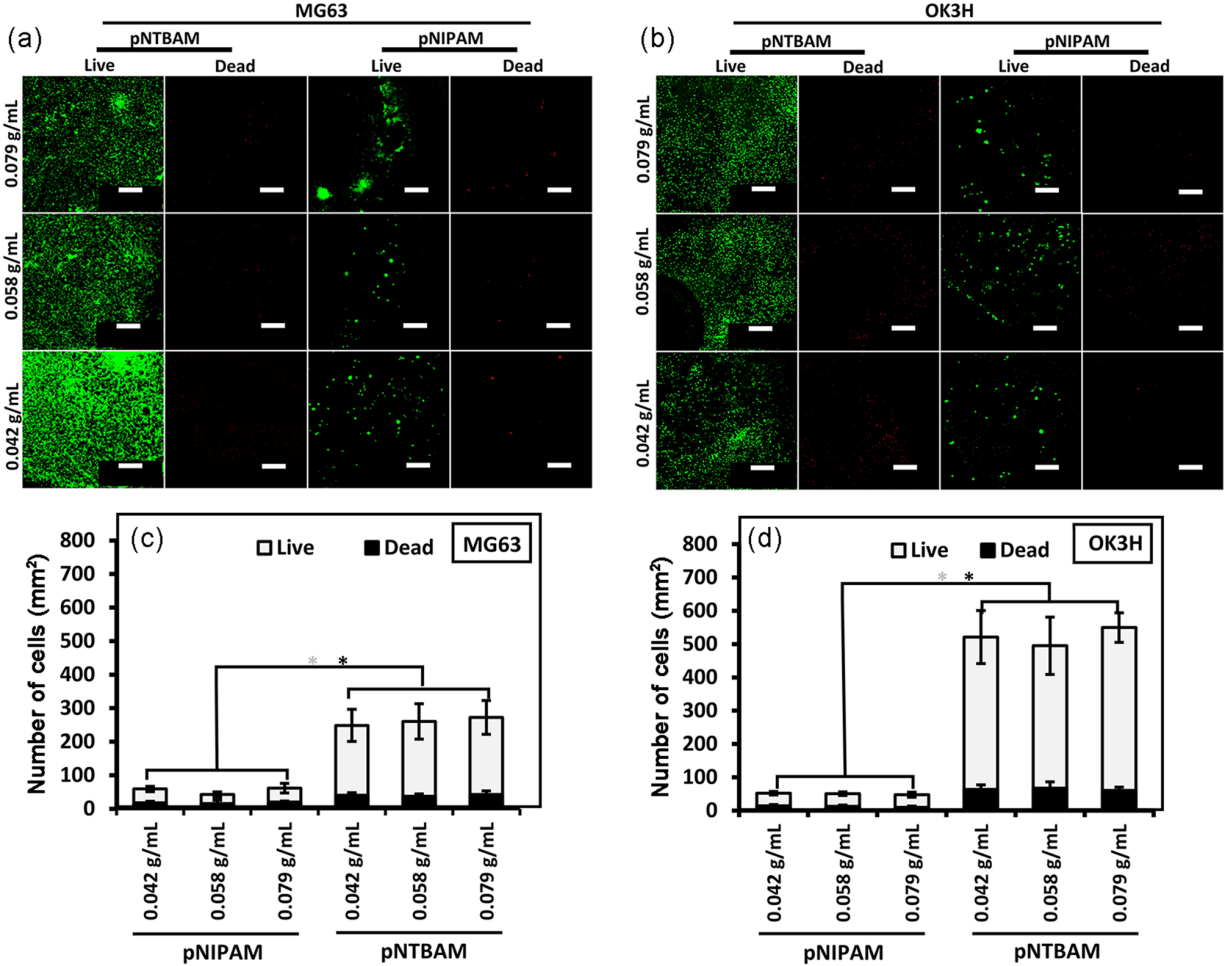
Survival and proliferation of MG63 and OK3H chondrocytes on hydrogels. **A**, **B** confocal images of live/dead stained MG63 and OK3H, respectively, seeded on hydrogel monomeric subgroups. Live cells (green) and dead cells (red) are presented. **C**, **D** Quantification of live (light colour bars) versus dead (dark colour bars) cells for MG63 and OK3H, respectively,per hydrogel (mm^2^) after 21 days in culture. Scale bar indicates 500 µm. Asterisk indicates significance at *P* ≤ 0.05. Data presented as mean ± SD, *n* = 3

Osteosarcoma MG63 and chondrogenic OK3H cells seeded onto pNIPAM samples formed distinct aggregates or clusters while conversely, with pNTBAM, they attach and spread out across the hydrogel surface (Fig. 4A, B). This scenario was concentration independent. There were consistently greater numbers of cells on pNTBAM versus pNIPAM hydrogels for both MG63 (pNTBAM 272.3 ± 50.2 cells/mm^2^ versus pNIPAM 61.4 ± 14.5 cells/mm^2^) (Fig. 4C) and OK3H (pNTBAM (550 ± 44.5 cells/mm^2^ versus pNIPAM 48 ± 7 cells/mm^2^) (Fig. 4D) with 0.079 g/mL seeded samples (*P* ≤ 0.05).

**Fig. 5:**
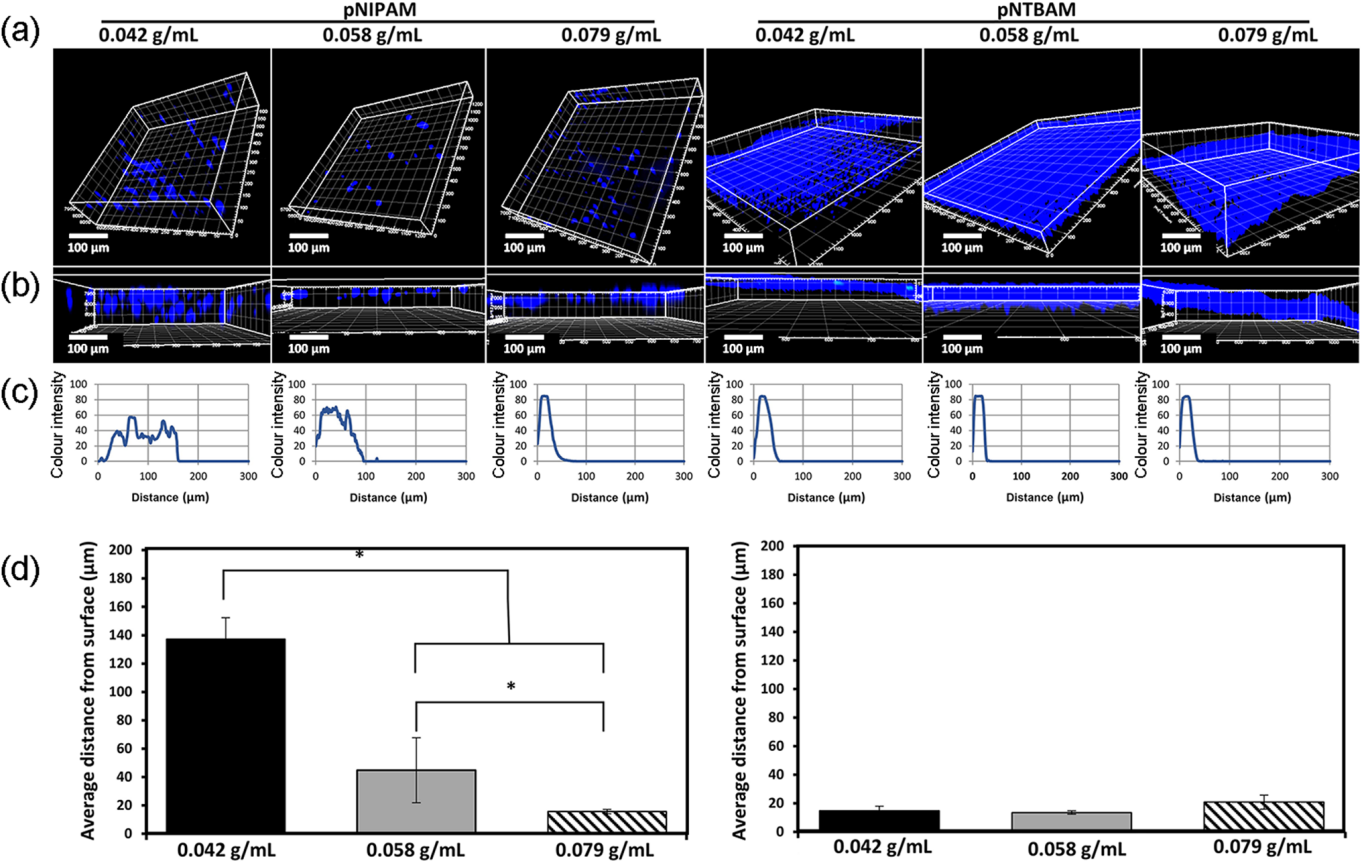
MG63 migration within hydrogel constructs. **A**, **B** Confocal 3D image volume reconstructions with DAPI-labelled nuclei (blue), viewed from surface (**A**) and the z-axis (**B**). Scale bar is 100 µm. **C** Quantification of DAPI colour intensity plotted against depth (µm from surface). **D** The average distance travelled by cells from hydrogel surface. Graphs in (**B**) are created by ImageJ software. Asterisk (*) indicates *P* ≤ 0.05. Data presented as mean ± SD, *n* = 3

We next sought to establish when hydrogels supported both surface cell adhesion and internal migration, being important for 3D construction of tissues in vitro. Irrespective of monomeric concentration little evidence of penetration was observed with pNTBAM as cells remained predominantly localised at the surface (Fig. 5A and B). This was similar to 0.079 g/mL pNIPAM. The maximal extent of migration was 15–20 µm below the surface with pNTBAM hydrogel samples, which was consistent with pNIPAM (0.079 g/mL). In contrast, migration into the remaining pNIPAM hydrogels was directly related to the monomeric concentrations used, with lowest values supporting migration to the greatest extent. Reduced monomeric concentrations supported more extensive migration with pNIPAM; 0.042 g/mL (137 ± 15 µm, *P* ≤ 0.05) and 0.058 g/mL (44.8 ± 19 pNIPAM µm) (Fig. 5C, D).

Taken together, our compression, porosity, viability, and migration data determined the selection of the specific hydrogel concentrations of 0.079 g/mL (pNTBAM) and 0.042 g/mL (pNIPAM) for primary cell experimentation. Further, based on identified properties (stiffer mass and high cellular density) pNTBAM was selected as a putative best choice for support of mineralization while the smaller pores, hydrophobicity, lower cellular density, cluster growth, and flexibility suggested pNIPAM as a putative choice for chondrogenic tissue growth.

### Hydrogels promote distinct osteogenic and chondrogenic behaviours

**Fig. 6:**
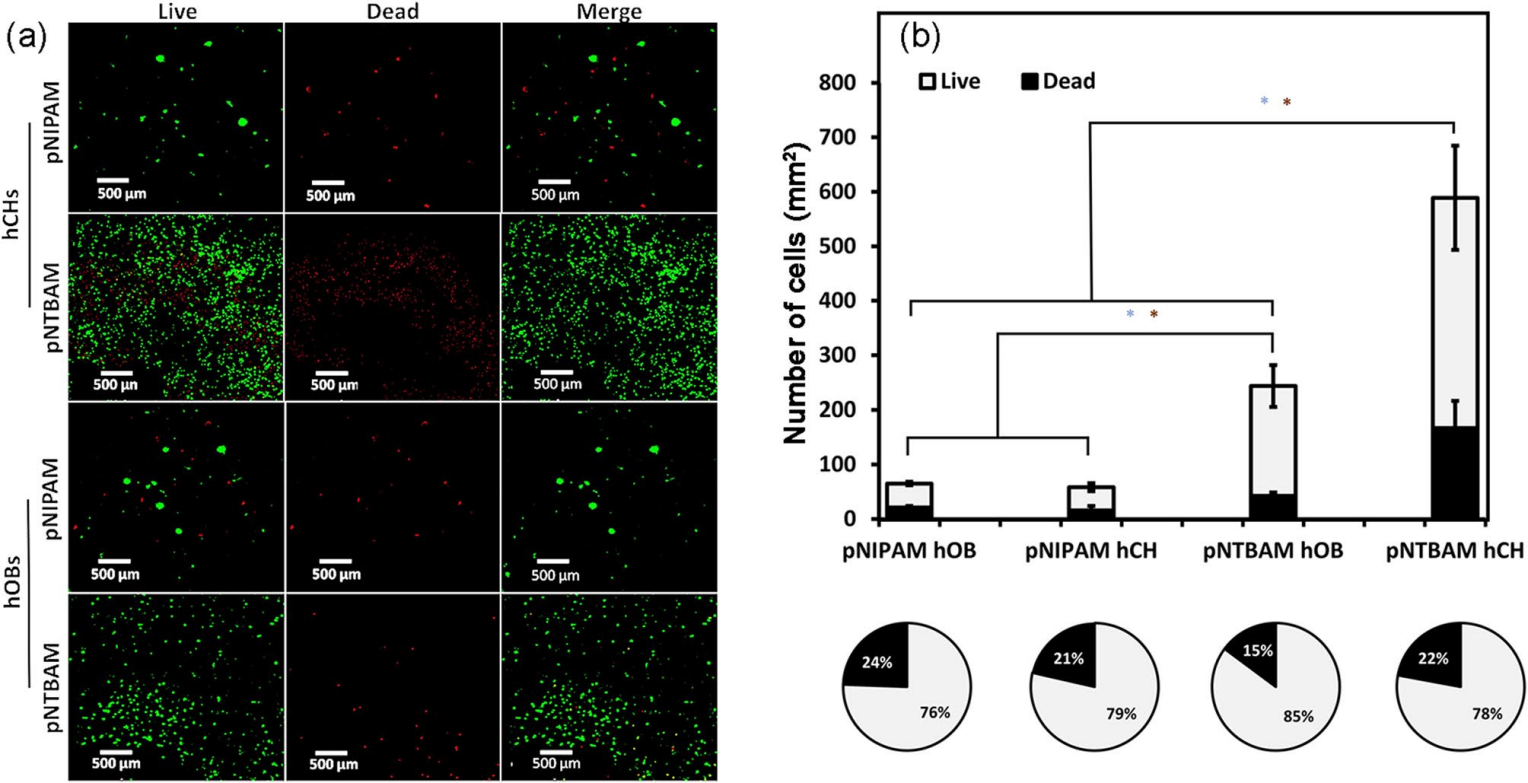
Survival and proliferation of primary osteoblast and chondrocytes on pNIPAM and pNTBAM. **A** Live (green), dead (red), and merged images are shown for each hydrogel with both hCHs and hOBs. Scale bar indicates 500 µm. **B** Live/dead quantification for primary hOBs and hCHs on hydrogel constructs. Data presented as cells per 1 mm^2^ of hydrogel surface. Pie charts indicate live/dead cells where the light segment corresponds to the percentage of live cells and the dark segment the percentage of dead cells. Asterisk indicates *P* ≤ 0.05. Data presented as mean ± SD, *n* = 3)

The viability of hOBs and hCHs seeded onto the selected hydrogel sub-types was assessed. Similar surface distribution patterns were apparent to those observed with MG63 and OK3H cell lines (Fig. 6A). Specifically, pNTBAM supported surface spreading while pNIPAM promoted tight, cluster, formation. Quantification of live/dead cell labelling indicated good viability across all conditions, with significantly greater levels of proliferation for hCHs (588 ± 95.7 cells /mm^2^) when compared to hOBs (243.6 ± 38.26 cells/ mm^2^) on pNTBAM (*P* ≤ 0.05). We again noted that pNIPAM displayed reduced cell numbers (58 ± 7.3 cells/mm^2^ for hOBs and 65 ± 3.3 cells/mm^2^) when compared to pNTBAM, (Fig. 6B). However, whilst proliferation levels were reduced, the overall percentages between live and dead cells were broadly consistent across both hydrogels and cell types (Fig. 6B).

**Fig. 7:**
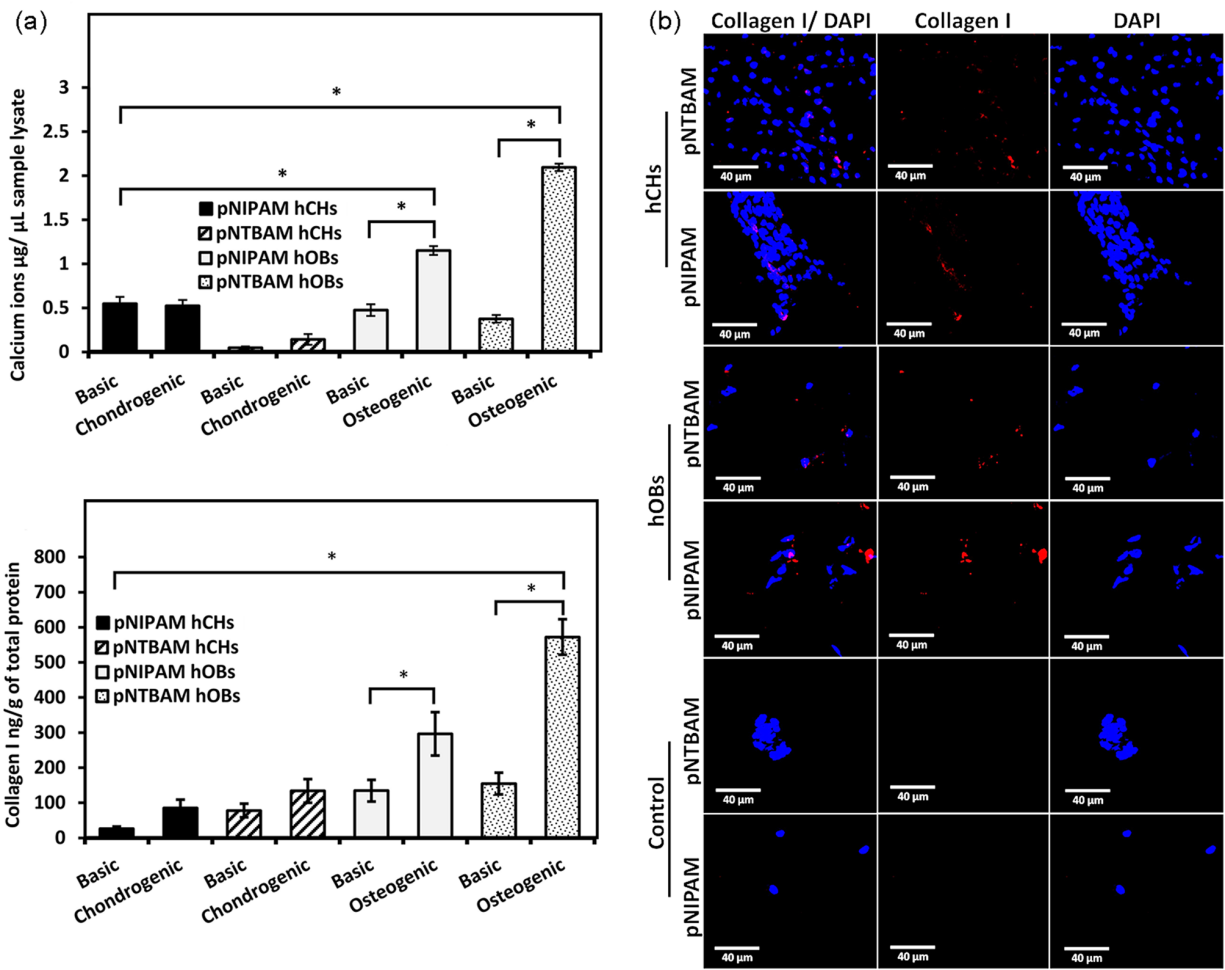
Osteogenic differentiation ofhOBs and hCHs on pNIPAM and pNTBAM. **A** Calcium ion quantification (Top Panel) for hOBs and hCHs seeded on pNIPAM and pNTBAM hydrogels and exposed to either basic, or osteogenic or chondrogenic differentiation induction media for 21 days. Elisa-based collagen I quantification (Bottom Panel) for samples as detailed above. Asterisk indicates *P* ≤ 0.05. Data presented as mean ± SD, *n* = 3). **B** Collagen I immunofluorescence and DAPI-labelled nuclei images captured by confocal microscopy. All images captured at 40× magnification. Scale bar indicates 40 µm

**Fig. 8:**
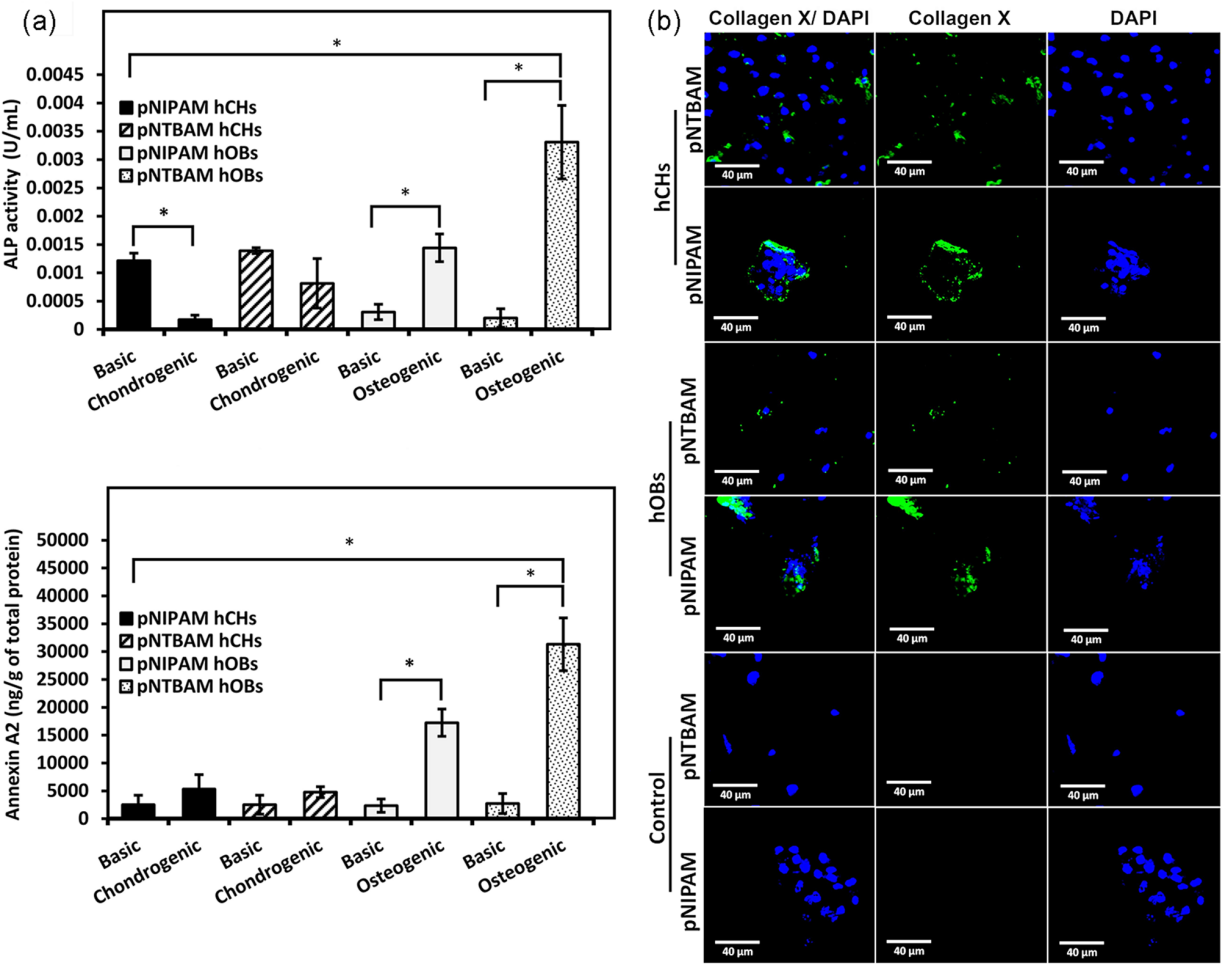
Mineralization activity of hOBs and hCHs on pNIPAM and pNTBAM hydrogels. **A** Bar figures comparing ALP activity (top panel) and annexin A2 expression (bottom panel) inhOBs and hCHs on pNIPAM and pNTBAM hydrogels and exposed to either basic, or osteogenic or chondrogenic differentiation induction media for 21 days. Asterisk indicates *P* ≤ 0.05. Data presented as mean ± SD, *n* = 3). **B** Collagen X immune-fluorescence and DAPI-labelled nuclei images captured by confocal microscopy. All images captured at 40× magnification. Scale bar indicates 40 µm

To explore the capacity of our selected hydrogel concentrations to support differentiation, primary cells were seeded onto hydrogels and exposed to either osteogenic or chondrogenic induction. hOB displayed elevated production ofcalcium ions, with both hydrogels, when maintained in osteogenic induction media when compared to their control samples (*P* ≤ 0.05). pNTBAM produced 2.09 ± 0.04 versus 0.37 ± 0.04 µg/µL calcium ions whilst pNIPAM produced the lower amount of 1.15 ± 0.04 versus 0.47 ± 0.06 µg/µL calcium ions (*P* ≤ 0.05). Collagen I levels produced by hOBs were significantly elevated in osteogenic media when compared to control samples (296 ± 61 versus 134 ± 30 ng/g of total protein for pNIPAM and 572 ± 50 versus 154 ± 30 ng/g of total protein for pNTBAM) (*P* ≤ 0.05) (Fig. 7A). This was observed to the greatest extent with pNTBAM (572 ± 50 ng/g of total protein) and lesser so with pNIPAM (296 ± 61 ng/g of total protein) (*P* ≤ 0.05). Complementary to increases in collagen I and calcium ion production we noted that ALP activity levels were increased from 0.2 × 10^3^ ± 0.1 × 10^3^ for controls to 3.3 × 10^3^ ± 0.6 × 10^3^ U/µL for pNTBAM osteogenic samples (*P* ≤ 0.05) which were themselves in turn significantly elevated (*P* ≤ 0.05) when compared to pNIPAM values of 1.4 × 10^3^ ± 0.2 × 10^3^ U/µL versus 0.3 × 10^3^ ± 0.1 × 10^3^ U/µL in untreated controls (*P* ≤ 0.05) (Fig. 8A).

**Fig. 9:**
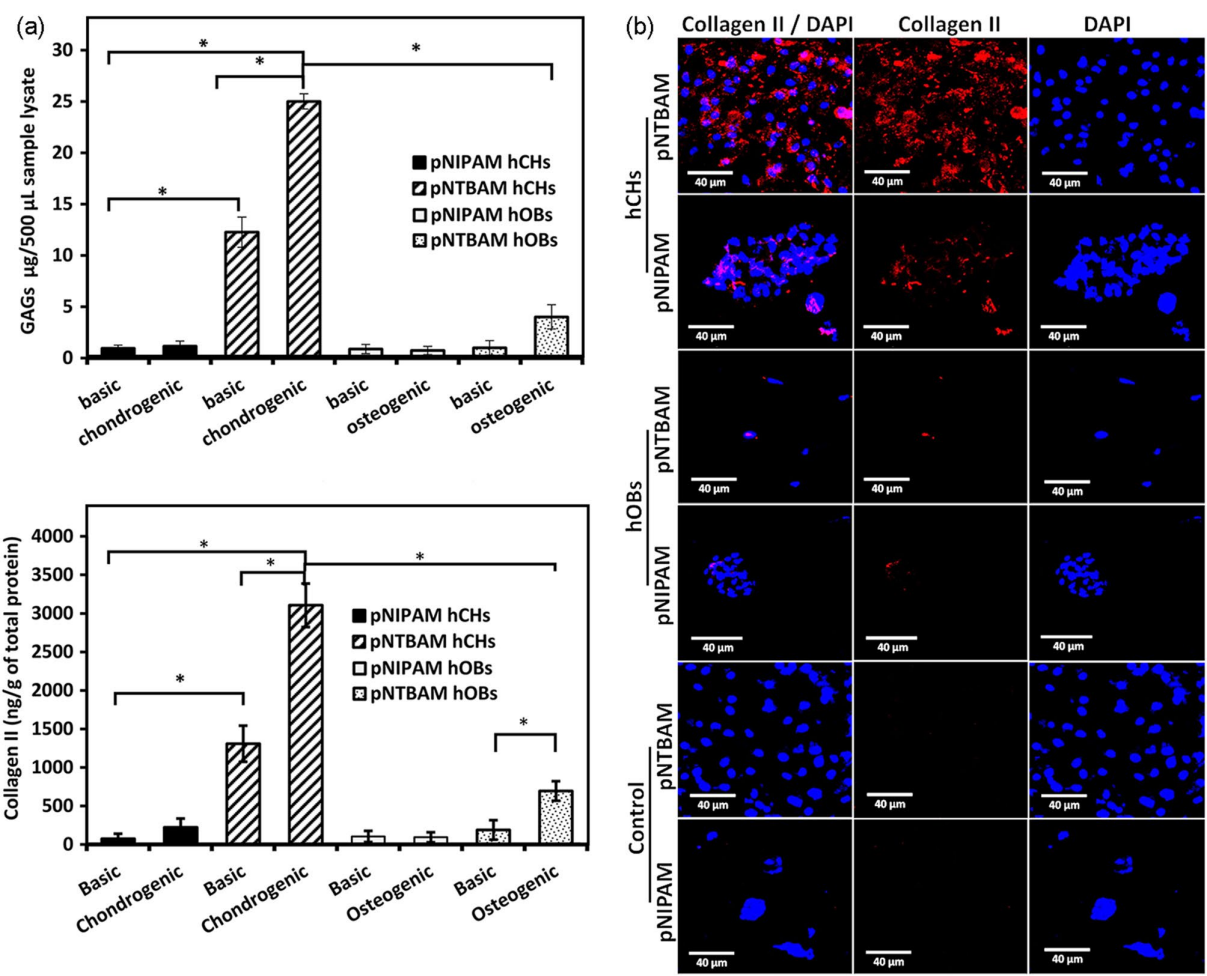
Chondrogenic differentiation on pNIPAM and pNTBAM. **A** GAG quantification of hCHs and hOBs seeded on hydrogel and incubated in either basic, osteogenic, or chondrogenic media after 21 days. Elisa-based collagen II quantification of samples (bottom panel) explained as above. Asterisk indicates *P* ≤ 0.05. Data presented as mean ± SD, *n* = 3. **B** Collagen X immunofluorescence and DAPI-labelled nuclei images captured by confocal microscopy. All images captured at 40× magnification. Scale bar indicates 40 µm

Annexin A2 displayed a similar pattern with significant elevation in both hydrogels compared to controls (*P* ≤ 0.05). pNTBAM produced 31.3 × 10^3^ ± 4.7 × 10^3^ in osteogenic compared to 2.7 × 10^3^ ± 1.8 ng/g control, whereas pNIPAM produced 17.2 × 10^3^ ± 2.4 × 10^3^ versus 2.3 × 10^3^ ± 1.2 × 10^3^ ng/g of controls (Fig. 8A). No evidence of chondrogenesis through GAG accumulation or collagen II expression was observed for hOB samples incubated in osteogenic media, irrespective of which hydrogel material was used (Figs. 9A).

Hydrogels seeded with hCHs, and maintained in chondrogenic media, displayed calcium ion levels comparable to controls and were significantly lower than hOBs samples maintained in osteogenic media (*P* ≤ 0.05) (Fig. 8A). However, evidence of calcium minerals was noted for hCHspNIPAM in both chondrogenic and control media where the later was significantly higher (*P* ≤ 0.05) when compared to pNTBAM (0.52 ± 0.06 µg/µL pNIPAM versus 0.14 ± 0.06 µg/µL for pNTBAM). Both were significantly lower (*P* ≤ 0.05) than hOB samples in osteogenic media. Hydrogels seeded with hCHs in chondrogenic media displayed increased collagen I levels when compared to controls (pNTBAM (85 ± 23 versus 26 ± 6 ng/g) and pNIPAM (133 ± 33 versus 78 ± 19 ng/g) (*P* > 0.05) (Fig. 5A). There was no significant change observed in levels of ALP activity in chondrogenic media supplemented hCHs on pNTBAM, whilst hCHs on pNIPAM showed significantly lower ALP activity in chondrogenic versus control samples. Chondrogenic hCHs hydrogel samples were significantly lower than osteogenic hOB hydrogel samples in all instances (*P* ≤ 0.05) (Fig. 8A).

Chondrogenic hCHs samples revealed significant elevation in GAG expression for pNTBAM hydrogels (25 ± 0.7 µg versus 12.26 ± 1.5 µg control) (*P* ≤ 0.05). pNIPAMhCHs samples were not significantly different compared to their controls and were significantly lower (*P* ≤ 0.05) than pNTBAM (1.15 ± 0.49 µg pNIPAM versus 25 ± 0.74 µg for pNTBAM chondrogenic samples).

Collagen II was significantly elevated in pNTBAM samples seeded with hCHs (3105 ± 282 ng/g versus 1309 ± 233 ng/g) (*P* ≤ 0.05). pNIPAM displayed no significant changes (224 ± 112 versus 74.8 ± 64.7 ng/g control). pNTBAM, again, showed significant elevation when compared to pNIPAM chondrogenic samples (3105 ± 282 versus 224 ± 112 ng/g for pNIPAM) (Fig. 8A).

Consistent with previous observations immune-labelling revealed strong collagen I expression with hOBs samples with lesser evident indication with hCHs samples (Fig. 6B). Collagen X was observed with both hOB and hCH irrespective of hydrogel (Fig. 7B). Differences in Collagen X expression were hydrogel, and not cell, specific with greater labelling present in pNIPAM. Collagen II expression was readily apparent with pNTBAM samples seeded with hCHs. Lesser expression was noted with hCHpNIPAM samples and little expression with hOB samples (Fig. 9B).

## Discussion

Poly(*N*-isopropylacrylamide) (pNIPAM) and poly(*N*-tert-butylacrylamide) (pNTBAM) hydrogels were produced by polymerization induced phase separation resulting from differing wettablity between monomer and polymer network, a widely applied method in the production of porous polymer scaffolds (Hutmacher 2001). Variation of monomer concentration and the solvent mixture can control pore size, with a thermodynamic demixing of the polymer-rich and solvent rich phases becoming more apparent as a the polymer network grows (Nam and Park 1999). The chemistry and stability of the monomer to be supported in the solvent phase is also important in determining the porous architecture of the final gel formed. A more hydrophobic monomer results in an increase in hydrophobicity of the polymer, resulting in earlier phase separation compared to a monomer of lower hydrophobicity at the same stage in polymer growth. This demixing again results in change in pore size and shape, and ultimately impacts on the macromechanical properties of the gel formed. In this work, reducing the monomeric concentration of polymers resulted in a significantly increased pore diameter for both hydrogels. The graded porosity of scaffolds, and resulting surface chemistry, are demonstrated to have distinct impacts on differentiation potential creating an opportunity for osteochondral construct generation (Lien et al. 2009; Jin et al. 2019).

Kaplan, et al. previously demonstrated the inverse relationship of polymer solution concentration and pore size further indicating a positive correlation between porosity and osteogenesis in the 50–100 µm pore size range (Karageorgiou and Kaplan 2005). Although highly porous, pNIPAM and pNTBAM hydrogels displayed an on average smaller pore size (a maximum average value of 35.7 µm ± 9 for pNIPAM and 21.2 ± 5 for pNTBAM) than those seen with cancellous bone (average of 300 µm) (Lee et al. 2012; Cooper et al. 2016). Our hydrogel porosity was comparable to that of the sub-chondral bone plate, which is a more compact layer with a smaller pore openings range from 20 to 30 µm in diameter (Bian et al. 2016).

Stiffness is an essential native property of tissue with impact on proliferation, orientation, and differentiation (Wells 2008). pNIPAM and pNTBAM revealed variable rigidity with 371 kPa for pNTBAM and 16 kPa for pNIPAM (Fig. 1B). The inherent hydrophobicity of pNTBAM accompanied by its limited porosity may be responsible for it having a more compact and stiffer composition than the highly porous hydrophilic pNIPAM (Cha et al. 2011). The cartilage compressive modulus is indicated to range from 200 to 500 kPa with region and cartilage layer dependence (Franz et al. 2001; Little et al. 2011) pNTBAM displayed a compressive modulus reflective of native cartilage. In contrast, neither hydrogel displayed values resembling the measured compressive modulus for bone tissues which can reach hundreds ofmegapascals (Pal 2014). However, material’s stiffness may not need to be identical to that of native tissue serving as a temporary matrix template (Yang et al. 2017).

Differences in the capacity of pNTBAM and pNIPAM to support cell proliferation were clear, with pNTBAM consistency presenter greater cell numbers for all cell types tested. Cell viability was, however, similar in all samples, with little cell death observed, (Figs. 4 and 6). pNIPAM hydrophilicity has potentially driven the formation of a more flexible, soft surface, promoting cell clustering without an increase in cell number (Tan et al. 2005). These observations are in agreement with previous studies that indicated the relationship between material stiffness, cell shape, proliferation, and attachment (Engler et al. 2004, 2006). Previous reports have evaluated the attachment of a transformed human cancer cell line on films of pNIPAM, pNTBAM, or a pNIPAM/pNTBAM copolymer, being consistent with our observations of enhanced attachment on pNTBAM regions. This is likely a result of the sterically hindered *tert*-butyl group disallowing presentation of N–H groups at the surface (Lynch et al. 2005) inhibiting protein adsorption and therefore restricting cell attachment.

Cell migration within hydrogel constructs is determined by porosity and internal architecture. Interconnected porous structures enable penetration of cells towards the core of the scaffold optimizing signalling communication (Sobral et al. 2011; Turnbull et al. 2018). Here, pNIPAM (0.042 g/mL) supported cell invasion and migration into the hydrogel interior (Fig. 4). In contrast, pNTBAM did not support cell penetration, irrespective of monomeric concentration (and therefore pore size distribution) presented. Hydrogel production through phase separation resulted in a lower monomeric concentration being associated with more water being incorporated between polymer phases when using a hydrophilic polymer i.e., pNIPAM. In contrast, the hydrophobic pNTBAM, coupled to the use of a methanol solvent, promoted an increased density with reduced water enclosure within the polymer part (Khoryani et al. 2018; Remanan et al. 2018).

In line with the main goal of creating an osteochondral scaffold, we examined primary cell differentiation capacity with pNIPAM and pNTBAM hydrogels. Both hydrogels promoted osteogenic behaviour, though elevated levels were observed with pNTBAM. Further, pNTBAM supported chondrogenic differentiation to a significantly greater extent than pNIPAM. These results indicate that a small change in chemistry on the monomer can cause a major change in biological responses of hydrogels formed. Here we have two polymers which could potentially be used together to support osteochondral tissue engineering promoting control over chondrogenic and osteogenic behaviour (Figs. 7 and 8).

Porosity may have impacted on osteogenic and chondrogenic differentiation which would support the elevated chondrogenic markers with pNTBAM when compared to pNIPAM. Di Luca et al. indicated that chondrogenic differentiation was guided by a smaller pore architecture (Di Luca et al. 2016a, b). This was determined by differentiation of human mesenchymal stem cells (hMSCs) on a gradient, poly(ethylene oxide terephthalate) and poly(butylene terephthalate, porous scaffold where cells displayed an increased chondrogenic behaviour, and GAG production, in the smaller pore gradient region (measured at an average of 326 µm). An earlier report from the same group identified that osteogenic differentiation, coupled with increased mineralization, was enhanced by a scaffold with a larger porous architecture (Di Luca et al. 2015). These features may partially clarify the variable tendencies for pNIPAM and pNTBAM to promote bone and cartilage cells, respectively, and how the distinctive polymers’ properties impacted their relevant biological performances. This supports the potential utility of both hydrogels in scaffold construction designed to mimic the osteochondral region.

## Conclusions

The pNIPAM and pNTBAM hydrogels investigated demonstrate variable characteristics in porosity and mechanical strength. These differences originated from chemical variation which accordingly resulted in hydrophilic, pNIPAM, and hydrophobic, pNTBAM features. These features impacted on cellular performance in several aspects including viability and specific cell function including differentiation. Mineralization was supported to variable extents with both but was more prevalent with pNTBAM. In addition, pNTBAM alone demonstrated biocompatibility (in terms of the fraction of live versus dead cells) with chondrogenic differentiation. We conclude that when taken together, these unique polymer characteristics create a potentially tuneable platform for 3D osteochondral scaffold generation for application in future tissue engineered complex repair situations.

## Data Availability

The datasets generated during the current study are available from the corresponding authors on reasonable request.
